# Health-related quality of life in older Chinese patients with diabetes

**DOI:** 10.1371/journal.pone.0229652

**Published:** 2020-02-27

**Authors:** Ye Zhuang, Qing-Hua Ma, Chen-Wei Pan, Jun Lu

**Affiliations:** 1 Department of Health Policy and Management, School of Public Health, Fudan University, Shanghai, China; 2 The 3rd People's Hospital of Xiangcheng District, Suzhou, China; 3 School of Public Health, Medical College of Soochow University, Suzhou, China; Qazvin University of Medical Sciences, ISLAMIC REPUBLIC OF IRAN

## Abstract

**Background:**

Although older diabetes patients with unique characteristics should be cared carefully to improve their health-related quality of life (HRQOL), the association between diabetes and HRQOL remain unclear, especially in Asians. We aimed to compare the HRQOL between older Chinese patients with type 2 diabetes (T2D) and their age-gender-matched controls.

**Methods:**

Older patients with T2D were recruited from a community hospital in Suzhou located in the east part of China while controls were selected from a community-based health survey of older adults aged 60 years or older. HRQOL of cases and controls was assessed by the EQ-5D-3L. The impact of T2D on HRQOL was investigated using a liner regression model and the relationship between T2D and EQ-5D health problems was evaluated using logistic regression models.

**Results:**

A total of 220 cases and 440 controls were included. The mean age of the participants was 68.8 years and women accounted for 69.1% of the study sample. The EQ-5D-3L index score was lower for older people with T2D (0.886) than their controls (0.955). After multivariable adjustment, the difference in ED-5D-3L index score between older people with and without T2D was 0.072. In logistic regression analyses, T2D was positively associated with reporting of problems in mobility (odds ratio [OR] = 5.00); pain/discomfort (OR = 1.66), and anxiety/depression (OR = 3.2).

**Conclusions:**

T2D has a detrimental effect on HRQOL of older Chinese people.

## Introduction

Diabetes has become one of the most common chronic diseases among older Chinese people (i.e. 60 years or older), with the prevalence being 20.4% [1] and more than 22.5% [2] in two major studies. It is estimated that more than 50% of patients with diabetes in China are above 60 years [3]. Given the robust increase in life expectancy and dramatic increase in the prevalence of unhealthy diet and lifestyle due to rapid economy growth and social development, the number of older peoples with diabetes will continue to increase in China.

Older patients with diabetes have several unique characteristics as older people are more vulnerable and susceptible to diseases. First, diabetes associates with an excess mortality risk [4] as well as a shorter life expectancy among older adults[5]. Second, older patients with diabetes often have a higher prevalence of late complications (e.g. retinopathy, nephropathy) due to the interaction effect between aging and hyperglycemia [6]. Third, older patients with diabetes usually experience substantial health conditions including comorbidities[7], poor functional status[8–10], chronic pain[11], and mental and emotional problems such as cognitive impairment[12] and depression[13]. Hence, diabetes in later life is a more serious problem than it in the early stage of life[14], and imposes a great disease and economic burden on healthcare system[15].

As diabetes can be controlled rather than cured, an important goal of healthcare towards older people is to improve their health-related quality of life (HRQOL) or retard the HRQOL deterioration in their remaining life years. Thus, assessing the HRQOL among older patients with diabetes is essential. Previous studies have shown that patients with diabetes have worse HRQOL in comparison with their peers of similar ages from general populations in Western countries [8–10, 16–18]. Two other studies have further provided and compared HRQOL weights (i.e. health utilities) between the two groups [17, 18], which is useful in cost-utility analysis for resource allocation decisions. However, reports on the association between diabetes and HRQOL in older Asian populations are limited, though a study in Japan has investigated this issue in younger adults aged 30 years or older [19].

There are various instruments measuring the HRQOL of diabetes patients[20, 21]. The EQ-5D is a widely used instrument based on utility, and its validity and reliability have been established[22]. In the present study, we aimed to evaluate the HRQOL of older patients with type 2 diabetes (T2D) by comparing HRQOL as measured by the EQ-5D between the patients and their age-gender-matched controls from a study of a general older population without diabetes in China. We also provided HRQOL weights for the two comparison groups.

## Methods

### Study participants

Outpatients with T2D were recruited from a community hospital in Suzhou, China, during the year 2014. The inclusion criteria were: 1) a physician diagnosis of T2D; 2) absence of cognitive impairment; and 3) aged18 years or older. T2D patients were asked not to eat any food after 8 pm the day before clinical examinations. After the clinical examination, consenting patients were invited to complete a paper-based questionnaire assessing their HRQOL using the EQ-5D-5L and EQ-5D-3L. Information regarding clinical and sociodemographic conditions was also collected. Of the 289 T2D patients aged 35 to 88 years recruited, 220 were no less than 60 years and were included in the present study.

Age- and gender- matched controls were chosen from a health survey on adults aged 60 years or older conducted in the same community in Suzhou during 2014. The study aimed to assess the HRQOL of older adults, and estimate the patterns, predictors and burden of common health outcomes of older people in eastern China[23]. Eligible participants (n = 5,614) were asked to complete a questionnaire collecting the information of their HRQOL and clinical and sociodemographic characteristics. The questionnaire is similar with that for T2D patients, except for two aspects: 1) HRQOL was only assessed using the EQ-5D-3L; 2) diabetes-related questions (e.g. diabetes complications) were not included.

Only the participants without diabetes were kept as study candidates for controls. Two subjects matching age and gender for each case were drawn randomly from the eligible participants without diabetes. As a result, a sample of 440 older people was used in the study as controls.

The study was conducted following the tenets of the Helsinki Declaration and was approved by the Institutional Review Board of Fudan University. All participants gave written informed consent at the recruitment stage of the study.

### Health-related quality of life

In the study, the HRQOL of older adults with and without diabetes was measured and valued by the EQ-5D-3L, which has demonstrated satisfactory validity and reliability in both general and patient populations in the mainland of China [24–26]. The EQ-5D-3L defines a respondent’s health status according to five dimensions: mobility, self-care, usual activities, pain/discomfort, and anxiety/depression. Assessment of anxiety/depression is the only psychometric property of the EQ-5D-3L. Within each dimension, there are three severity levels corresponding to no, moderate, or extreme problems. An individual’s response to the five dimensions jointly form a health state which can be assigned a HRQOL weight score (i.e. EQ-5D-3L index score) to indicate the value of the health state. The index score used in the study was from the EQ-5D-3L value set elicited from the general Chinese population, which ranges from -0.149 to 1.0, with negative values, 0, and 1.0 indicating health states worse than being dead, being dead, and full health, respectively [27].

### Covariates

The health characteristics of older adults with T2D were collected from both survey and clinical examinations. Data on diabetes complications, chronic conditions other than diabetes (e.g. lung disease, arthritis, depression/anxiety, cancer, and heart disease), and age at onset of diabetes were collected through a survey. The health characteristics of controls were obtained from the survey only, which inquired whether they have a set of common chronic conditions in older adults. Sociodemographic characteristics including age, gender, education level, and working status for both populations were self-reported in the survey.

### Statistical analysis

Data collected from older adults with and without T2D were pooled for analysis. The sociodemographic/health characteristics and HRQOL of the two groups were compared using chi-square tests for categorical variables and student’s t-test for continuous variables. We fitted multivariate regression models to explore the impact of T2D on HRQOL controlling for covariates. First, the EQ-5D index score was analyzed using a linear regression model. Second, the relationship between the presence of EQ-5D health problems and T2D was investigated using separate logistic regression models. The covariates entered into the multivariate models included education level (no formal education vs. formal education), working status (working or looking after work vs. retired), obesity (presence vs. absence), and chronic conditions other than diabetes (presence vs. absence).

## Results

A total of 220 older patients with T2D (cases) and 440 participants without T2D (controls) from the general older population were included in the study. Their characteristics are shown in Table 1. Compared to controls, cases were more likely to be working or looking after work, obese, and with chronic conditions other than diabetes.

**Table 1:** Characteristics of the study participants.

Characteristics	Cases (n = 220)	Age- and gender- matched controls (n = 440)	P-value
**Gender**			
**Female**	152 (69.1)	304 (69.1)	
**Male**	68 (30.9)	136 (30.9)	
**Age, mean(SD)**	68.6 (6.6)	68.6 (6.6)	
**Education level**			
**No formal education**	124 (56.4)	250 (56.8)	0.89
**Formal education**	96 (43.6)	190 (43.2)	
**Working status**			
**Working or looking after work**	105 (47.7)	132 (30.0)	<0.0001
**Retired**	115 (52.3)	308 (70.0)	
**BMI level**			
**<25**	141 (64.1)	361 (82.0)	<0.0001
**≥25**	79 (35.9)	79 (18.0)	
**Chronic conditions other than diabetes**			
**Yes**	183 (83.2)	336 (76.4)	0.04
**No**	37 (16.8)	104 (23.6)	
**Fasting blood glucose (mmol/L)**			
**<7.0**	109 (49.5)		
**≥7.0**	111 (55.5)		
**Duration of diabetes (years), Mean (SD)**	7.4(5.3)		

T2D: type 2 diabetes; SD: standard deviation; BMI: body mass index.

Overall, cases had worse HRQOL than controls. The mean ± standard deviation of EQ-5D index score of cases (0.886 ± 0.17) was significantly lower than that of controls (0.955 ± 0.07, p <0.001). In addition, cases were more likely to experience problems in all EQ-5D dimensions (Table 2). The prevalence of having problems in each of the five dimensions ranged from 5.5% (self-care) to 37.3% (pain/discomfort) among cases and from 0.9% (usual activities) to 24.1% (pain/discomfort) among controls, respectively (Table 2).

**Table 2:** Comparison of the EQ-5D index score and health problems between elderly patients with T2D and age- and gender- matched general population.

	Cases	Age- and gender- matched controls	P-value
**EQ-5D index score, Mean (SD)**	0.886 (0.17)	0.955 (0.07)	<0.0001
**EQ-5D health problems, N (%)**			
**Mobility**	39 (17.7)	18 (4.1)	<0.0001
**Self-care**	12 (5.5)	5 (1.1)	<0.0001
**Usual activities**	21 (9.6)	4 (0.9)	<0.0001
**Pain/discomfort**	82 (37.3)	106 (24.1)	0.0004
**Anxiety / depression**	41 (18.6)	28 (6.4)	<0.0001

T2D: type 2 diabetes; SD: standard deviation.

The results of multivariate regression analysis comparing the EQ-5D index score and health problems between the two groups are shown in Table 3. After adjustment for covariates, the EQ-5D score of cases was 0.072 point lower than that of controls (95% confidence interval [95% CI]: 0.048, 0.105). Significant associations were observed between having T2D and reporting of problems in all three EQ-5D dimensions analyzed, with the odds ratios (ORs) being 5.0 (95% CI: 2.22, 11.28) for mobility, 1.66 (95% CI: 1.15, 2.40) for pain/discomfort, and 3.20 (95% CI: 1.61, 6.37) for anxiety/depression, respectively. Two dimensions (i.e. self-care and usual activities) were not analyzed as a low proportion of general older participants reported such problems (0.9% for usual activities and 1.1% for self-care).

**Table 3:** Multivariate analysis of the EQ-5D index and health problems between elderly patients with T2D and age- and gender- matched general population.

	Age- and gender- matched general population	Elderly with T2D	(95% CI)
**EQ-5D index score**	**Ref.**	-0.072	(-0.105, -0.048)
**EQ-5D health problems**	**Odds ratio**	**Odds ratio**	
**Mobility**	1	5.00	(2.22, 11.28)
**Pain/discomfort**	1	1.66	(1.15, 2.40)
**Anxiety/depression**	1	3.20	(1.61, 6.37)

T2D: type 2 diabetes.

## Discussion

In this case-control study, we compared the HRQOL between older patients with T2D and their age- gender-matched peers using the EQ-5D-3L self-report questionnaire. We found that T2D has a detrimental effect on the HRQOL among older Chinese adults as it leads to a higher probability of suffering problems in mobility, pain/discomfort, and anxiety/depression.

The finding that older patients with T2D had a significant HRQOL deficit is consistent with the finding from previous studies[8–10, 16–18]. The difference in mean EQ-5D-3L index score between the two groups (0.072) is also comparable to the difference (0.059) reported in a study in Poland [18]. On the other hand, the HRQOL weights of our study seemed to be higher. We reported that the mean EQ-5D scores were 0.886 and 0.955 for cases and controls while the scores were 0.739 and 0.798 in the Poland study and within a range (0.7, 0.8) in a study in UK[17]. The higher values of our study could be explained by two reasons. First, the Chinese EQ-5D-3L value set generates higher values. Indeed, a study using Japanese value set which also yields higher EQ-5D-3L values for older patients with diabetes: 0.88 and 0.82 for T2D patients aged from 60 to 69, and 70 years and over, respectively [28]. Second, both cases and controls in our study reported fewer health problems in all EQ-5D dimensions compared to their corresponding peers in the UK and Poland studies. For example, the prevalence of reporting any problems in EQ-5D dimensions of older T2D patients ranged from 5.5% (self-care) to 37.3% (pain/discomfort) in our study, and 53.2% (self-care) to 84.7% (mobility) in the study in Poland. This may be partially because only outpatients with T2D were enrolled and such patients generally reported relatively favorable HRQOL. Alternatively, it may imply better HRQOL of T2D patients in China. Given the significant difference in characteristics (e.g. onset of diabetes, diet structure, genetic factors) between East Asians with T2D and those from Western countries [29], future studies are warranted to investigate the association between these characteristics and HRQOL.

T2D results in higher frequency in reporting problems in all the three dimensions (mobility, pain/discomfort, and anxiety/depression) analyzed in this study. The findings are in line with the other studies based on the EQ-5D-3L[17, 18], and similar with the findings based on other HRQOL instruments[8–10, 16]. Previous studies have consistently indicated that older people with diabetes reported lower physical function in comparison to those without[8–10]. This observation might result from various aspects of functional impairment including peripheral neuropathy and vascular disease, as well as balance problems caused by diabetes and its complications[14]. Older patients with diabetes are more likely to experience pain. The increased pain report among the patients may be due to a decreased pain threshold which may be a direct consequence of hyperglycemia as the infusion of glucose to normal individuals has been found to impair the ability to tolerate pain, despite the concurrence of peripheral neuropathy[30]. The occurrence of depression is also found to be more frequent in older patients with diabetes[14]. Evidence has suggested a bi-directional relationship between diabetes and depression[31]. Depression in people with diabetes may result from psychosocial stressors of having a chronic condition. In addition, hypoglycemia is able to induce negative mood states in patients with diabetes[31].

These problems not only impair the HRQOL of older patients with diabetes, but also relate to various bad outcomes[14]. Among older people with diabetes, depression is strongly related to hospitalization and mortality; chronic pain is in relation to suicide attempts; and mobility impairment is associated with increased falls and injuries. The joint effects of aging and diabetes make the healthcare of older patients with diabetes extremely difficult. The patients in China may face more challenges as the diabetes care in China is far from satisfactory[32]. Hence, healthcare professionals should pay particular attention to the treatment and management of diabetes in older people with diabetes. Furthermore, joint efforts will be needed between government and healthcare providers to deliver higher standards of care. Improving the HRQOL of older patients with diabetes is now a great challenge from a public health prospective. Current evidence has indicated that improving medication adherence[33] and self-care behaviors[34] might be effective. Efforts should be made towards identifying potential meditators such as religious coping and social support in the relationship between diabetes and HRQOL[33].

The study had three major limitations. First, only outpatients were recruited in the study, and nobody had severe complications such as foot ulcer and amputation. Hence, future studies using a representative sample of older patients with diabetes are warranted. Second, patients with diabetes were recruited form a hospital while controls were from a community population, which may have biased the results. Nevertheless, the hospital is located within the community, which could largely decrease the potential selection bias. Third, there were few participants who reported “extreme problems”in the survey and the study sample was apparently healthy. Thus, the generalizability of the present study's findings may not reach to severe patients.

## Conclusion

This study showed that older people with T2D had worse HRQOL than controls from the general population. The HRQOL weight of the patients provided by this study may be useful in economic evaluation of healthcare interventions for the diabetes patients in China.

## Supporting information

S1 Dataset (SAV)
